# A systematic review and meta-analysis of transthoracic echocardiogram vs. cardiac magnetic resonance imaging for the detection of left ventricular thrombus

**DOI:** 10.1093/ehjimp/qyad041

**Published:** 2023-12-07

**Authors:** YuZhi Phuah, Ying Xin Tan, Sheref Zaghloul, Sharmaine Sim, Joshua Wong, Saba Usmani, Lily Snell, Karish Thavabalan, Carmen Lucia García-Pérez, Niraj S Kumar, Hannah Glatzel, Reubeen Rashid Ahmad, Luciano Candilio, Jonathan J H Bray, Mahmood Ahmed, Rui Providencia

**Affiliations:** University College London Medical School, 74 Huntley St, London WC1E 6DE, UK; University College London Medical School, 74 Huntley St, London WC1E 6DE, UK; Department of Cardiology, Royal Free Hospital, London, UK; University College London Medical School, 74 Huntley St, London WC1E 6DE, UK; University College London Medical School, 74 Huntley St, London WC1E 6DE, UK; University College London Medical School, 74 Huntley St, London WC1E 6DE, UK; University College London Medical School, 74 Huntley St, London WC1E 6DE, UK; University College London Medical School, 74 Huntley St, London WC1E 6DE, UK; University College London Medical School, 74 Huntley St, London WC1E 6DE, UK; National Medical Research Association, London, UK; Stoke Mandeville Hospital, Aylesbury, UK; Brighton And Sussex Medical School, Brighton, UK; University College London Medical School, 74 Huntley St, London WC1E 6DE, UK; Department of Cardiology, Royal Free Hospital, London, UK; University College London Medical School, 74 Huntley St, London WC1E 6DE, UK; Oxford Heart Centre, John Radcliffe Hospital, Oxford, UK; Department of Cardiology, Royal Free Hospital, London, UK; University College London Medical School, 74 Huntley St, London WC1E 6DE, UK

**Keywords:** left ventricular thrombus, echocardiography, cardiac magnetic resonance, diagnosis, screening, sensitivity and specificity

## Abstract

Transthoracic echocardiography (TTE) is the most commonly used imaging modality to diagnose left ventricular thrombus (LVT), however, cardiac magnetic resonance (CMR) remains the gold standard investigation. A comparison of the diagnostic performance between two modalities is needed to inform guidelines on a diagnostic approach towards LVT. We performed a systematic review and meta-analysis to investigate the diagnostic performance of three methods of TTE (non-contrast, contrast, and apical wall motion scoring) for the detection of LVT compared to CMR as a reference test. Studies comprising 2113 patients investigated for LVT using both TTE and CMR were included in the meta-analysis. For non-contrast TTE, pooled sensitivity and specificity were 47% [95% confidence interval (CI): 32–62%], and 98% (95% CI: 96–99%), respectively. In contrast, TTE pooled sensitivity and specificity values were 58% (95% CI: 46–69%), and 98% (95% CI: 96–99%), respectively. Apical wall motion scoring on non-contrast TTE yielded a sensitivity of 100% [95% CI: 93–100%] and a specificity of 54% (95% CI: 42–65%). The area under the curve (AUC) values from our summary receiver operating characteristic curve (SROC) for non-contrast and contrast TTE were 0.87 and 0.86 respectively, with apical wall motion studies having the highest AUC of 0.93. Despite high specificity, routine contrast and non-contrast TTE are likely to miss a significant number of LVT, making it a suboptimal screening tool. The addition of apical wall motion scoring provides a promising method to reliably identify patients requiring further investigations for LVT, whilst excluding others from unnecessary testing.

## Lay summary

The formation of left ventricular thrombus (LVT), a blood clot in the left pumping chamber of the heart, can lead to serious complications such as a stroke. Whilst cardiac magnetic resonance (CMR) is the best imaging tool to detect these clots, the most used tool is a transthoracic echocardiogram (TTE), which visualizes the heart by placing an ultrasound on the chest. This is due to the affordability and widespread availability of TTE. Thus, it is important to know how TTE compares to CMR when it comes to detecting LVT.

This study pools the results of previous research to compare the diagnostic performance of three different methods of TTE compared to CMR for detecting LVT.

1. Non-contrast TTE.
2. Contrast TTE: The addition of an enhancing dye is thought to improve imaging.
3. Apical wall motion scoring: Evaluating the movement of the heart’s walls using TTE.

Our results show that current methods of TTE may miss half of the patients with LVT and that the use of contrast did not provide significant improvement. Interestingly, the use of apical wall motion scoring was able to accurately detect all the patients with LVT. This shows promise as a future tool to reliably exclude patients from unnecessary testing, whilst identifying those who need further investigations.

## Graphical Abstract

**Graphical abstract:**
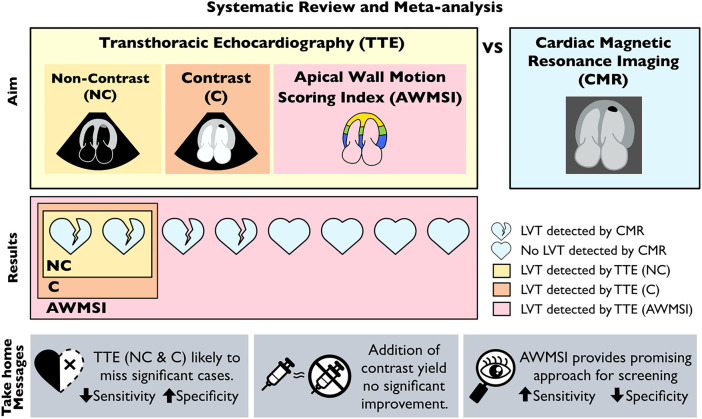

## Introduction

Left ventricular thrombus (LVT) is an important complication of cardiac disorders such as severe ischaemic and non-ischaemic cardiomyopathy, and is associated with a significant risk of embolic events such as stroke:^1^ 2.5% to 15% of patients with acute myocardial infarction (AMI) develop LVT. There is a significantly increased risk of major adverse cardiovascular events at one year in LVT vs. non-LVT patients (36% vs. 5.8%) driven by systemic embolization and stroke (20% in LVT vs. 2.1% in non-LVT patients).^2^ The detection of LVT is therefore critical for the management of patients with cardiac diseases, as it can help guide the management of antithrombotic treatment to improve patient outcomes.

Two imaging modalities commonly used for the detection of LVT are transthoracic echocardiography (TTE) and cardiac magnetic resonance imaging (CMR). Key imaging characteristics of LVT in both CMR and TTE are summarized in Supplementary data online, *Figure S1*. TTE is widely available, non-invasive, and has a relatively low cost, while CMR offers superior spatial resolution and tissue characterization.^3,4^ Non-contrast echocardiography is the most used imaging modality to detect LVT, and its use is recommended in guidelines by the European Society of Cardiology and the American Heart Association.^5,6^ Additionally, widespread use of CMR is impractical due to the limited availability of the technique alongside significant costs. However, TTE has a lower sensitivity than CMR (when surgical or pathological validation of LVT is used as the index test).^7^ This brings into question whether TTE can serve as an alternative to CMR as a diagnostic tool, and how clinicians should determine who to refer to CMR.

There have been several studies comparing the diagnostic accuracy of TTE vs. CMR for the detection of LVT.^7–9^ Notably, a previous meta-analysis of studies published before May 2020^8^ reported TTE as a reasonable alternative to CMR. However, this study was limited by the fact that results from both contrast and non-contrast TTE were combined, making it difficult to interpret the findings. The use of echocardiography-enhancing contrast agents has been recommended to increase the sensitivity of LVT detection, but evidence of its diagnostic utility is mixed.^6^ Additionally, whilst calculation of the wall motion score index is classically used to investigate left regional contractile dysfunction,^10^ apical wall motion scoring applied to routine non-contrast TTE has been proposed as a potential screening test for LVT^11^ [infographic on Supplementary data online, *Figure S1*]. Therefore, a comprehensive assessment of the diagnostic performance of these three applications of TTE compared to CMR as a reference is needed.

This paper provides an updated meta-analysis comparing the diagnostic performance of non-contrast and contrast TTE vs. CMR separately for the detection of LVT in patients with cardiac disease. We also further investigate the diagnostic performance of apical wall motion scoring vs. CMR for the detection of LVT. The results of this meta-analysis will help inform future guidelines on the diagnostic algorithm for LVT and hopefully will bring attention to the need for more primary research on this important topic.

### Highlights

1. Non-contrast and contrast TTE has high specificity compared to CMR {98% [95% confidence interval (CI): 96–99%]} but is likely to miss a significant number of LVT due to low sensitivity [47% (95% CI 32–62%) and 58% (95% CI: 46–69%) respectively].
2. This study did not find significant improvement in sensitivity and specificity of contrast TTE compared to non-contrast TTE.
3. The addition of apical wall motion scoring to routine non-contrast TTE is a promising approach to reliably screen for patients who require further investigations for LVT, with a sensitivity and specificity value of 100% (95% CI: 93–100%), and 54% (95% CI: 42–65%), respectively.

## Methods

In this study, we aimed to evaluate the diagnostic yield of LVT using various forms of TTE, including contrast, non-contrast, and apical wall motion scoring, compared to CMR as a reference standard. We followed the Preferred Reporting Items for Systematic Reviews and Meta-Analyses (PRISMA) guidelines to conduct a systematic review and meta-analysis of original research studies.^12^

The primary outcome of interest was the sensitivity and specificity of TTE compared to CMR for the detection of LVT. Additionally, we examined secondary diagnostic accuracy measures such as the area under curve (AUC) value of the summary receiver operating characteristic (SROC) curve, diagnostic odds ratio (DOR), and positive and negative likelihood ratios (LR+ and LR–).

### Eligibility criteria

The following are the inclusion criteria for the relevant studies:

1. Studies including adult patients who are at high risk of developing LVT, such as those with left ventricular ejection fraction <50%, or patients post-MI.
2. The study must assess thrombosis in the left ventricle.
3. Studies evaluating the diagnostic yield of TTE compared to CMR.
4. The study must report the sensitivity and specificity of the diagnostic test.
5. Study is an original work written in English.

Any studies that do not meet the above criteria, such as case reports, commentaries, and review articles will be excluded.

### Information sources and search strategy

We conducted a comprehensive search of the Ovid Medline and EMBASE databases from 1 January 1960 to 1 May 2023. Details on our search strategy can be seen in Supplementary data online, *Table S1*. Additionally, we reviewed the reference lists of relevant meta-analyses and review papers to identify potential articles.

### Study selection process

Rayyan was utilized to manage and organize the selected abstract and article information. To ensure a thorough selection process, five independent investigators (Y.P., Y.T., J.W., S.S., and S.U.) conducted a screening of study titles and their abstracts, with a minimum of two investigators screening each study. Eligibility assessment was made by a minimum of two investigators on the full texts from the studies that passed screening. In instances where discrepancies arose, consensus was reached through a discussion with other co-authors (Y.P. and Y.T.).

### Data collection process

Data collection involved extracting diagnostic test accuracy parameters for three different modalities of TTE in comparison to CMR. Specifically, contrast TTE, non-contrast TTE, and apical wall motion studies on non-contrast TTE were analysed. From each study, four key parameters were collected for TTE vs. CMR in diagnosing LVT: (i) true positive, (ii) false positive, (iii) true negative, and (iv) false negative values. These values were then reported along with sensitivity and specificity results. Additional data points, including the type of CMR imaging utilized as a reference, the time interval between TTE and CMR, and patient demographics such as age and male percent (%) were collected. Patient data on cardiovascular risk factors (hypertension, hypercholesterolaemia, diabetes mellitus, and tobacco use) and medications (aspirin, beta-blockers, angiotensin-converting enzyme inhibitors/angiotensin receptor blockers, statins, and loop diuretics) was also collected. All data was collected using a standardized proforma, and reviewed by at least two independent reviewers. In cases where certain data was not reported in a study, it was assumed to be missing at random.

### Risk of bias and applicability

The Quality Assessment of Diagnostic Accuracy Studies 2 (QUADAS-2) was used to evaluate the risk of bias and applicability of included studies.^13^ Signalling questions were tailored to our study, and two independent reviewers assessed all included studies using the tool. In cases of discrepancies, a consensus was reached through discussion with other co-authors.

### Synthesis of results

We conducted three separate meta-analyses for non-contrast TTE, contrast TTE, and apical wall motion scoring. Quantitative analysis was performed using R studio (Version 2022.12.0+353). For the meta-analysis, the metaprop function was used, whilst meta-regression analysis was performed using the metareg function. A random effects model was used to conduct our univariate analysis. A forest plot with corresponding 95% confidence intervals (95% CI) was used to represent the sensitivities and specificities of each modality. The I^2^ statistic was used to determine between-study heterogeneity. We defined the thresholds for low, moderate, and high levels of heterogeneity with an I^2^ value of 25%, 50%, and 75% respectively.^14^ The reitsma function was used for our bivariate analysis, which implements the Reitsma model, demonstrated by Harbord et al 2007 to be equivalent to the hierarchical SROC model of Rutter and Gatsonis 2001.^15–17^ This was done to obtain an SROC curve, which illustrates the DOR and accuracy of the tests. This model accounted for both within- and between-study heterogeneity. A weighted average and standard deviation were used to present pooled patient data.

### Heterogeneity analysis

We conducted a sensitivity analysis on the non-contrast and contrast TTE group using the leave-one-out study approach to explore the sources of heterogeneity in study outcomes. Furthermore, we performed a subgroup analysis on non-contrast TTE studies using meta-regression to identify potential contributors to heterogeneity. The subgroup analysis was based on three factors: (i) CMR type, (ii) indication for LVT imaging, and (iii) study population size.

## Results

### Study selection

The abstract screening was performed on 306 unique citations. Subsequently, 22 studies were assessed via full-text screening. A total of 11 studies were included in our quantitative analysis (see Supplementary data online, *Figure S2*).

### Baseline characteristics

In the pooled population of 2113 patients from included studies, the mean age was 58.2 ± 12.8 years, with a 78.0 ± 9.3% male predominance. Time between imaging modalities was reported to be within 7 days for all studies, with six studies performing both within 24 h of each other. The pooled cohort included patients investigated for LVT using TTE and CMR, indicated due to severe heart failure or following AMI. Study characteristics can be found in *Table 1*, with results of individual studies in Supplementary data online, *Table S2*. Patient data on cardiovascular risk factors and medication history are presented in *Table 2*. Ten studies with 2019 patients were included for the analysis of non-contrast TTE vs. CMR, whilst the contrast TTE vs. CMR group included four studies with 542 patients. Two studies comprising of 275 patients were included for apical wall motion scoring on non-contrast TTE.

**Table 1:** Study characteristics

Study, year	Patients (*n*)	Age ± std	Male %	Population^a^	Reference standard	Technique used^b^	Time between imaging modalities
Weinsaft 2016^11^	201	56 ± 12	84.1	A	DE (delayed enhancement)-CMR	1 + 2	24 h
Weinsaft 2009^3^	121	61.2 ± 13.3	76.9	B	DE-CMR	1 + 2	Within 7 days
Weinsaft 2011^18^	243	60 ± 15	63.4	C	DE-CMR	1	Within 7 days
Delewi 2012^19^	194	56.1 ± 9.4	84.9	A	cine-CMR	1	24 h
Garg 2012^20^	481	N/R	N/R	B	CE (contrast enhanced)-CMR	1 + 2	Within 7 days
Meurin 2015^21^	78	59.1 ± 12.1	72	A	DE-CMR	1	24 h
Sürder 2015^22^	113	56.8 ± 10.2	88.3	A	DE-CMR	1	24 h
Chaosuwannakit 2021^23^	206	60.2 ± 14.2	68.4	C	DE-CMR	1	
Kim 2017^24^	74	54 ± 11	91.2	A	DE-CMR	3	24 h
Phan 2019^25^	210	N/R	85	A	DE-CMR	1	Within 7 days
Kim 2014^26^	192	N/R	N/R	A	DE-CMR	1 + 2	24 h

^a^A = post-MI, B = mixed (e.g. post-MI, heart failure, stroke). C = left ventricular systolic dysfunction.

^b^1 = non-contrast TTE, 2 = contrast TTE, 3 = apical wall motion scoring. Data that was not reported (N/R) in the primary paper was assumed to be missing at random.

**Table 2:** Demographical characteristics

Study	Risk factors (%)				Medication history (%)				
	Hypertension	Hypercholesterolaemia	Diabetes Mellitus	Tobacco use	Aspirin	Beta-blockers	ACE/angiotensin receptor blockers*	Statins	Loop diuretic
Weinsaft 2016^11^	43.8	49.8	23.4	N/R	99.0	95.5	58.7	97.0	6.0
Weinsaft 2009^3^	66.9	88.4	33.1	33.1	85.1	77.7	66.9	79.3	16.5
Kim 2017^24^	47.3	45.9	24.3	27.0	97.3	98.6	73.0	94.6	N/R
Weinsaft 2011^18^	61.3	38.7	35.4	23.9	57.6	51.4	45.7	N/R	31.3
Delewi 2012^19^	27.8	19.6	6.2	46.9	96.4	N/R	N/R	N/R	N/R
Garg 2012^20^	N/R	N/R	N/R	N/R	N/R	N/R	N/R	N/R	N/R
Meurin 2015^21^	33.0	8.0	20.0	43.0	100.0	100.0	99.0	99.0	N/R
Sürder 2015^22^	41.8	41.2	10.7	58.2	97.3	91.1	85.7	99.1	33.9
Chaosuwannakit 2021^23^	N/R	N/R	N/R	N/R	76.2	N/R	N/R	N/R	N/R
Phan 2019^25^	47.1	45.7	19.5	57.6	N/R	94.3	82.9	97.6	7.1
Kim 2014^26^	N/R	N/R	N/R	N/R	N/R	N/R	N/R	N/R	N/R

N/R = data not reported in primary paper.

### Risk of bias and applicability

Of the 11 studies included, nine were found to be of low risk of bias. Sürder^22^ was classified as high risk because operators of TTE were not blinded to CMR results. Garg^20^ and Chaosuwannakit^23^ were classified as unclear risk, as information relevant to our risk of bias assessment was unavailable. A tabular representation of the results of our QUADAS2 assessment is in Supplementary data online, *Table S3*.

### Results of synthesis

#### Non-contrast TTE vs. CMR

**Figure 1:**
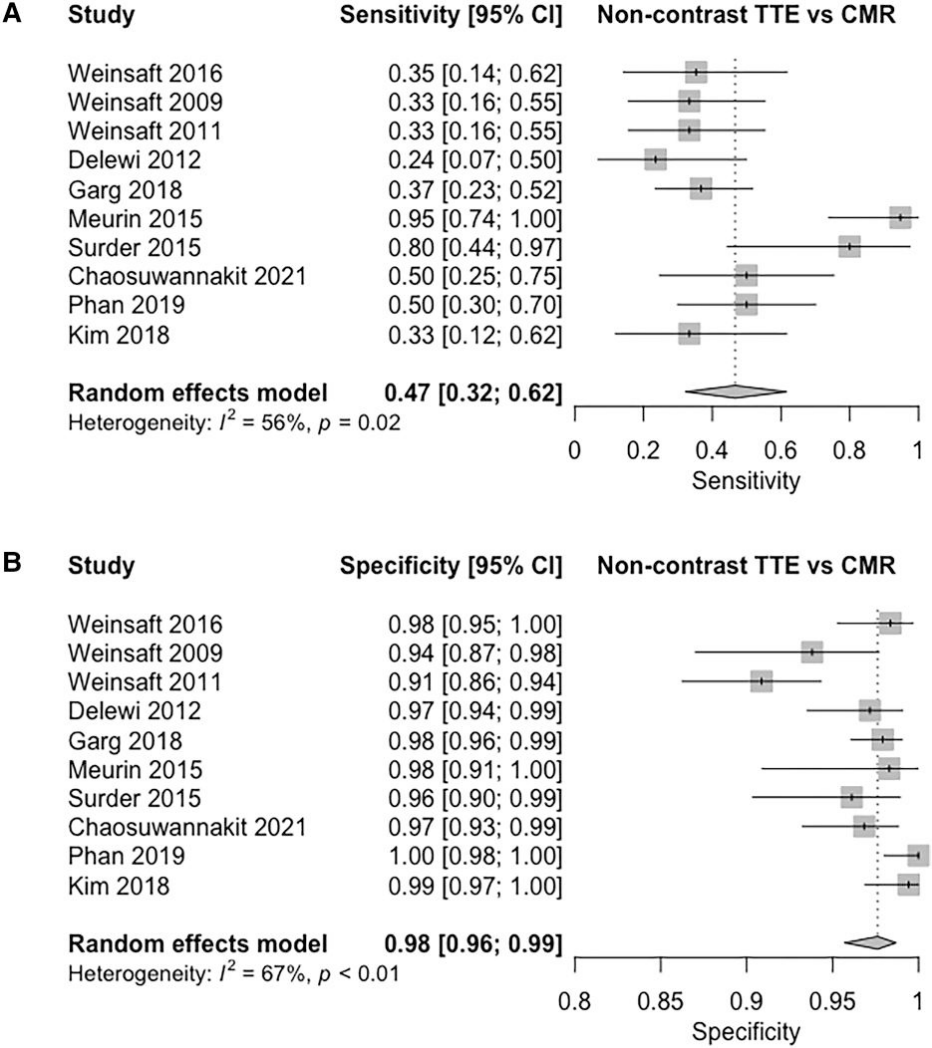
(*A*) Sensitivity of non-contrast TTE for the detection of LVT compared to CMR (*B*) specificity of non-contrast TTE for the detection of LVT compared to CMR.

In our meta-analysis of non-contrast TTE vs. CMR (10 studies), pooled sensitivity and specificity values were 47% (95% CI 32–62%, I^2^ = 56%, *P* = 0.02; *Figure 1A*), and 98% (95% CI: 96–99%, I^2^ = 67%, *P* < 0.01; *Figure 1B*), respectively. Pooled DOR was 24.8 (95% CI, 11.6–46.9), with pooled LR+ and LR– being 14.0 (95% CI, 7.63–23.5) and 0.58 (95% CI, 0.47–0.69), respectively. The shape of the bivariate SROC curve (see Supplementary data online, *Figure S3A*) and an AUC of 0.87 suggest good discriminative capacity.

#### Contrast TTE

**Figure 2:**
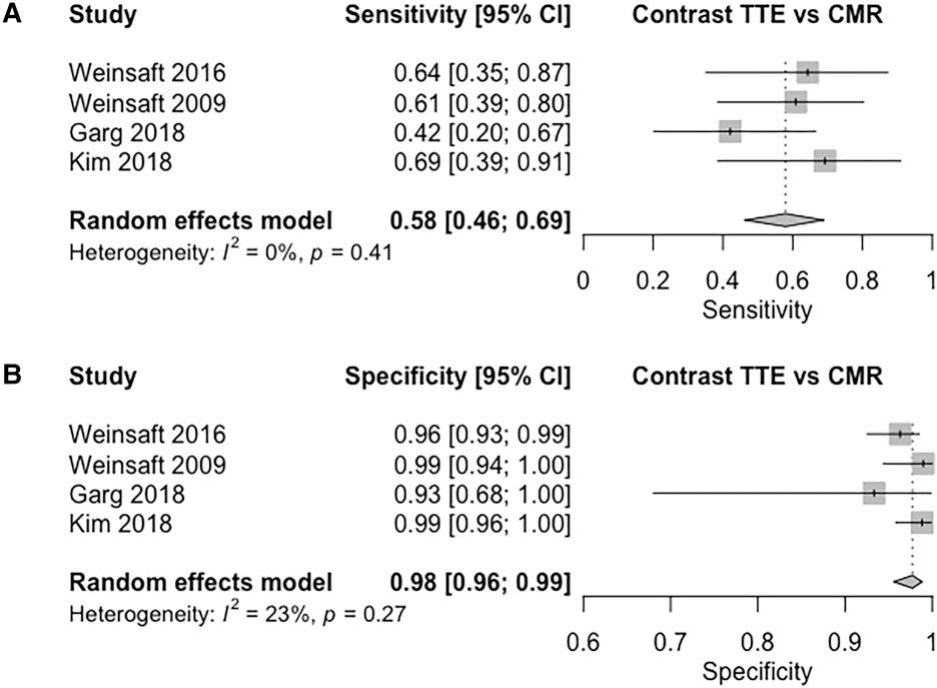
(*A*) Sensitivity of contrast TTE for the detection of LVT compared to CMR (*B*): specificity of contrast TTE for the detection of LVT compared to CMR.

Pooled comparison of contrast TTE vs. CMR (four studies) showed pooled sensitivity and specificity values of 58% (95% CI: 46–69%, I^2^ = 0%, *P* = 0.41; *Figure 2A*), and 98% (95% CI: 96–99%, I^2^ = 23%, *P* = 0.27; *Figure 2B*), respectively. Pooled DOR was 60.9 (95% CI: 15.6–165), with pooled LR+ and LR– being 24.5 (95% CI, 8.34–56.6) and 0.43 (95% CI, 0.30–0.58), respectively. The shape of the bivariate SROC curve (see Supplementary data online, *Figure S3B*) and an AUC of 0.86 suggest similar discriminative capacity compared to non-contrast TTE.

#### Apical wall motion studies applied to non-contrast TTE

**Figure 3:**
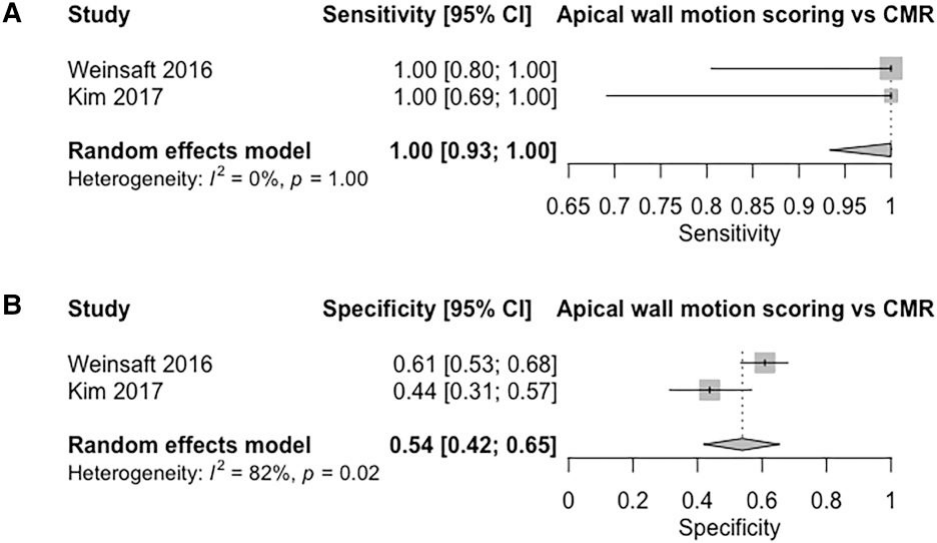
(*A*) Sensitivity of apical wall motion scoring on non-contrast TTE for the detection of LVT compared to CMR (*B*) specificity of apical wall motion scoring on non-contrast TTE for the detection of LVT compared to CMR.

Meta-analysis comparing the diagnostic yield of apical wall motion scoring applied to non-contrast TTE vs. CMR (2 studies) showed a sensitivity and specificity values of 100% (95% CI: 93–100%, I^2^ = 0%, *P* = 1.00; *Figure 3A*), and 54% (95% CI: 42–65%, I^2^ = 82%, *P* = 0.02; *Figure 3B*) respectively. Pooled DOR was 60.3 (95% CI, 3.15–299), with pooled LR+ and LR– being 2.08 (95% CI, 1.39–3.12) and 0.11 (95% CI, 0.01–0.45), respectively. The shape of the bivariate SROC curve (see Supplementary data online, *Figure S3C*) and an AUC of 0.93 suggest better discriminative capacity than both contrast and non-contrast TTE despite its lower specificity.

### Investigation of heterogeneity

#### Sensitivity analysis for non-contrast and contrast TTE

A leave-one-out analysis for non-contrast TTE vs. CMR (see Supplementary data online, *Table S4*) resulted in pooled sensitivity ranging from 39–49%, whilst pooled specificity had a small range of 97–98%. Exclusion of Sürder,^22^ which had a high risk of bias, resulted in a pooled sensitivity and specificity of 43% (95% CI: 30–58%, I^2^ = 48%) and 98% (95% CI: 96–99%, I^2^ = 71%), which was not significantly different to the original pooled effect sizes. Whilst the exclusion of this study resulted in a lower I^2^ value, heterogeneity was still considered to be at moderate to borderline-high levels. Similarly, exclusion of studies of unclear risk of bias Garg^20^ and Chaosuwannakit^23^ did not significantly impact the effect size or heterogeneity of both sensitivity and specificity for non-contrast TTE. Meurin *et al.* ^21^ was the greatest contributor of heterogeneity in sensitivity results, with its exclusion leading to an I^2^ of 21% along with the greatest decrease in pooled sensitivity at 39% (95%CI: 33–46%). Our sensitivity analysis on contrast TTE (see Supplementary data online, *Table S5*) resulted in pooled sensitivity from 55 to 64%, and specificity from 97 to 99%. Interestingly, the exclusion of Garg *et al*.,^20^ which had an unclear risk of bias, led to a sensitivity of 64% [95% CI: 50–76%]. Thus, whilst pooled sensitivity for contrast and non-contrast TTE was not significantly different, the exclusion of outlier studies such as Meurin *et al.*^20^ and Garg *et al*.^21^ led to a significant improvement in the pooled sensitivity of contrast TTE compared to non-contrast TTE.

#### Subgroup analysis

The indication for LVT imaging was found to have a significant impact on specificity for non-contrast TTE. Studies investigating non-contrast TTE vs. CMR in post-AMI patients were more likely to yield higher specificity results (coefficient = 1.4, *P* < 0.05) compared to other indications. No significant results were found for the remaining subgroups. In all subgroups, the test for residual heterogeneity was significant (*P* < 0.05). Further subgroup analysis with patient cardiovascular risk factors and medication use was not done due to missing data.

## Discussion

### Summary of evidence

To the best of our knowledge, this is the first meta-analysis evaluating the diagnostic yield of three different modalities of TTE for the diagnosis of LVT. Our assessment is important to inform future guidelines on the diagnostic approach for LVT, where TTE is used as a potential screening tool to stratify patients requiring further investigations. We aim to build upon a previous meta-analysis by performing the analysis of contrast and non-contrast TTE studies separately, as well as investigating apical wall motion scoring as a potential screening tool for LVT.^8^

Whilst both non-contrast and contrast TTE studies showed very high pooled specificity of 98% (95% CI: 96–99%) for LVT compared to CMR, they suffer from low pooled sensitivity values [47% (95% CI: 32–62%) and 58% (95% CI: 46–69%), respectively]. This suggests that both non-contrast and contrast TTE is likely to miss approximately half of LVT in patients. Interestingly, contrast TTE did not significantly improve sensitivities for LVT compared to non-contrast TTE. Similarly, there were no significant differences between the pooled DOR, LR+, and LR– values of contrast and non-contrast TTE studies. Additionally, bivariate SROC curves and AUC values for contrast and non-contrast TTE (0.86 and 0.87, respectively) indicates similar discriminative capacity.

The use of contrast-echocardiography, especially post-AMI, is not without its risks. Contrast-echocardiography with Sonovue is contraindicated in patients with recent acute coronary syndrome, or in patients with clinically unstable ischaemic heart disease.^27^ Current recommendations by the American Heart Association on the diagnosis of LVT support the use of contrast TTE to improve diagnostic sensitivity for LVT.^6^ Our study suggests that the use of contrast TTE provides limited utility for the diagnosis of LVT in terms of sensitivity and specificity.

In studies applying apical wall motion scoring to non-contrast TTE, pooled sensitivity compared to CMR was significantly higher than routine contrast and non-contrast studies at 100% (95% CI: 93–100%). However, pooled specificity was significantly reduced at 54% (95% CI: 42–65%). This suggests that despite the risks of falsely diagnosing patients with LVT, apical wall motion scoring on non-contrast TTE may reliably exclude those without LVT. This was reflected in reduced LR+ and LR–values, whilst no significant difference was seen in the DOR. Bivariate analysis of apical wall motion studies yielded the highest AUC (0.93) of the three modalities investigated. Differences in specificity results for apical wall motion studies seen between Weinsaft *et al*.^11^ (cut-off score of ≥5) and Kim *et al*.^24^ (cut-off score of ≥3) may be attributed to the different cut-off scores used for diagnosis of LVT. However, we were unable to do a subgroup analysis to further investigate this due to the limited number of primary studies on apical wall motion scoring and LVT detection.

LVT is typically identified on TTE through direct visualization of the echo-dense mass, which most likely contributes to the high specificity seen in our analysis.^28^ However, it suffers from poor sensitivity, possibly due to poor image quality or smaller LVT size.^29^ The addition of apical wall motion scoring on routine non-contrast TTE removes the need to directly visualize LVT and provides a promising approach to reliably stratify patients at risk of LVT and exclude patients from unnecessary further testing. A diagnostic approach using apical wall motion scoring for LVT following AMI was recently proposed by Camaj *et al*.^29^ A potential approach in LVT diagnosis could incorporate the diagnostic criteria of routine TTE studies with the apical wall motion cut-off score for risk stratification. This may provide high specificity when LVT can be diagnosed through direct visualization, whilst utilizing the high sensitivity of apical wall motion scoring to identify individuals who may require further investigations with CMR for LVT that are not directly visible via TTE.

The prognostic utility of using apical wall motion scoring as a screening tool for LVT has yet to be evaluated.^30^ A cohort study comparing embolic outcomes between patients who had LVT detected on CMR but not echocardiography vs. LVT detected by both found no significant difference in the cumulative incidence of embolic events.^30^ Thus, more studies are needed to investigate if the usage of apical wall motion scoring would significantly improve clinical outcomes of patients whose LVT would have been missed on routine TTE. Additionally, due to inconsistencies in the cut-off scores used, it is unclear as to what degree of apical wall motion abnormality should be considered a significant risk factor to the development of LVT. According to the wall motion scoring index (see Supplementary data online, *Figure S1*), a single segment akinesia or dyskinesia would have a score of 3 and 4, respectively, which would have met the threshold for Kim *et al*. 2017 but not Weinsaft *et al*. 2011.^7,10,11,18,24,31^ Thus, further studies are required to further understand how different degrees of severity for apical wall motion abnormalities are associated with an increased risk of developing LVT.

Whilst apical wall motion scoring displayed 100% sensitivity compared to CMR, it is important to consider that TTE methods can be limited by suboptimal acoustic windows, which is an inherent limitation compared to using CMR in LVT diagnosis. In the context of this analysis, none of the two studies investigating apical wall motion scoring reported any patient exclusions due to suboptimal acoustic windows.^11,24^ However, given the relatively small sample size of this analysis, further research ensuring to include patients despite suboptimal acoustic windows may yield lower sensitivity values seen thus far. Nonetheless, whilst apical wall motion scoring was never likely to match CMR as a gold standard imaging test for LVT, the issue of acoustic windows should not detract from its potential as a promising screening test compared to routine contrast and non-contrast TTE.

Our sensitivity analysis found that most of the heterogeneity in the sensitivity and specificity of non-contrast TTE results could be attributed to one or two outlier studies. It also suggests that the lack of significant difference in the pooled diagnostic performance between contrast and non-contrast studies may be attributed to outlier studies. However, the reasons for such different results are uncertain. Additionally, whilst our subgroup analysis suggests that specificity in non-contrast TTE improved for post-AMI patients, the reasons for this are unclear. Thus, further studies are needed to clarify sources of heterogeneity seen here and to verify if contrast TTE truly provides diagnostic benefit over non-contrast TTE.

### Limitations

The main limitation of our study is the small number of primary studies included in the analysis for contrast TTE and apical wall motion studies, thus reducing the power and certainty of our results. Whilst there are other existing studies on the imaging of cardiac thrombus, many did not meet the inclusion criteria detailed above. For example, some studies did not perform a direct comparison between TTE and CMR on the same patient or may have failed to report values such as the sensitivity and specificity for LVT diagnosis. More primary studies would be needed to confidently identify how contrast TTE affects diagnostic performance, as well as testing for apical wall motion studies to understand whether such high sensitivity levels can be replicated. Additionally, due to inconsistency in patient demographic data reported in each study, we were unable to perform a subgroup analysis due to the missing data. Increased reporting of baseline characteristics within these studies would have enabled a more comprehensive subgroup analysis.

Another limitation was the lack of anatomical reference standards from cardiac surgery or forensic examination, as well as the pooling of studies using different forms of CMR as a reference standard. However, our subgroup analysis based on the CMR modality used as a reference in the studies included did not find a significant difference between modalities. Despite this, a future analysis with more primary studies is required to verify this.

## Conclusions

Both contrast and non-contrast TTE have good specificity compared to CMR but are suboptimal as a screening test to reliably exclude patients without LVT. The addition of contrast was not found to improve diagnostic performance compared to non-contrast TTE, suggesting reduced utility in clinical practice. Apical wall motion scoring applied to non-contrast TTE has lower specificity, yet it represents a promising screening tool to identify patients requiring further investigations. Further studies are encouraged to validate the findings of our analysis, which may involve investigating contrast and non-contrast TTE alongside apical wall motion scoring for the diagnosis of LVT in a large patient population.

## Supplementary Material

qyad041_Supplementary_Data

## Data Availability

The data underlying this article was derived from the following articles available in the public domain.^3,4,11,19–26^

## Lead author biography

YuZhi Phuah is a final year medical student at University College London. He has a keen interest in clinical research and has previously co-authored published works on urology and medical education. Currently, he is working closely with clinicians in cardiology to write systematic reviews and meta-analyses on novel cardiovascular topics. YuZhi is always enthusiastic about new opportunities to participate and learn more about clinical research and methodology.
