# Supplementary material for: A systematic review and meta-analysis of transthoracic echocardiogram vs. cardiac magnetic resonance imaging for the detection of left ventricular thrombus
Source: Eur Heart J Imaging Methods Pract. 2023 Dec 7;1(2):qyad041. doi: 10.1093/ehjimp/qyad041 (PMC11240154; doi:10.1093/ehjimp/qyad041)
Supplement: qyad041_Supplementary_Data [file qyad041_Supplementary_Data.zip › Supplementary Tables.docx]

| 1. “left ventricle” OR “left ventricular” 2. “thrombus” OR “thrombi” 3. “CMR” OR “cardiac magnetic resonance” 4. “TTE” OR “echocardiography” OR “echocardiogram” 5. “detect*” OR “diagnose*” OR “assess*” OR “screen*” | 1. #1 AND #2 AND #3 AND #4 AND #5 2. Limit 6 to English language 3. Limit 7 to human 4. Remove duplicates from 8   Items found = 306 |
| --- | --- |

Supplementary Table 1: Search strategy

| **Non-contrast TTE Studies** | **TP** | **FN** | **FP** | **TN** |
| --- | --- | --- | --- | --- |
| Weinsaft 2016 (11) | 6 | 11 | 3 | 181 |
| Weinsaft 2009 (3) | 8 | 16 | 6 | 91 |
| Weinsaft 2011 (27) | 8 | 16 | 20 | 199 |
| Delewi 2012 (29) | 4 | 13 | 5 | 172 |
| Garg 2012 (19) | 18 | 31 | 9 | 423 |
| Meurin 2015 (21) | 18 | 1 | 1 | 58 |
| Sürder 2015 (18) | 8 | 2 | 4 | 99 |
| Chaosuwannakit 2021 (20) | 8 | 8 | 6 | 184 |
| Phan 2019 (30) | 13 | 13 | 0 | 184 |
| Kim 2014 (31) | 5 | 10 | 1 | 76 |

| **Contrast TTE Studies** | **TP** | **FN** | **FP** | **TN** |
| --- | --- | --- | --- | --- |
| Weinsaft 2016 (11) | 9 | 5 | 7 | 183 |
| Weinsaft 2009 (3) | 14 | 9 | 1 | 96 |
| Garg 2012 (19) | 8 | 11 | 1 | 14 |
| Kim 2014 (31) | 9 | 4 | 2 | 169 |

| **Apical wall motion scoring studies** | **TP** | **FN** | **FP** | **TN** |
| --- | --- | --- | --- | --- |
| Weinsaft 2016 (11) | 17 | 0 | 72 | 112 |
| Kim 2017 (23) | 10 | 0 | 36 | 28 |

Supplementary Table 2: Diagnostic parameters for individual studies (TP = true positive, FN = false negative, FP = false positive, TN = true negative)

| STUDY | RISK OF BIAS | | | | APPLICABILITY CONCERNS | | |
| --- | --- | --- | --- | --- | --- | --- | --- |
|  | PATIENT SELECTION | INDEX TEST | REFERENCE STANDARD | FLOW AND TIMING | PATIENT SELECTION | INDEX TEST | REFERENCE STANDARD |
| Weinsaft 2016 (11) | Low | Low | Low | Low | Low | Low | Low |
| Kim 2017 (23) | Low | Low | Low | Low | Low | Low | Low |
| Weinsaft 2009 (3) | Low | Low | Low | Low | Low | Low | Low |
| Weinsaft 2011 (27) | Low | Low | Low | Low | Low | Low | Low |
| Delewi 2012 (29) | Low | Low | Low | Low | Low | Low | Low |
| Garg 2012 (19) | Unclear | Unclear | Unclear | Unclear | Low | Low | Low |
| Meurin 2015 (21) | Low | Low | Low | Low | Low | Low | Low |
| Sürder 2015 (18) | Low | High | Low | Low | Low | Low | Low |
| Chaosuwannakit 2021 (20) | Low | Low | Low | Unclear | Low | Low | Low |
| Phan 2019 (30) | Low | Low | Low | Low | Low | Low | Low |
| Kim 2014 (31) | Low | Low | Low | Low | Low | Low | Low |

Supplementary Table 3: Tabular representation of QUADAS-2 results

| Left-out Study | Sensitivity | | Specificity | |
| --- | --- | --- | --- | --- |
|  | Sensitivity [95% CI] | I^2^ | Specificity [95% CI] | I^2^ |
| Weinsaft 2016 (11) | 0.48 [0.32; 0.65] | 0.61 | 0.98 [0.95; 0.99] | 0.67 |
| Weinsaft 2009 (3) | 0.49 [0.32; 0.65] | 0.60 | 0.98 [0.96; 0.99] | 0.70 |
| Weinsaft 2011 (27) | 0.49 [0.32; 0.65] | 0.60 | 0.98 [0.97; 0.99] | 0.11 |
| Delewi 2012 (29) | 0.49 [0.34; 0.65] | 0.56 | 0.98 [0.95; 0.99] | 0.69 |
| Garg 2012 (19) | 0.48 [0.32; 0.65] | 0.60 | 0.98 [0.95; 0.99] | 0.62 |
| Meurin 2015 (21) | 0.39 [0.33; 0.46] | 0.21 | 0.98 [0.95; 0.99] | 0.70 |
| Sürder 2015 (18) | 0.43 [0.30; 0.58] | 0.48 | 0.98 [0.96; 0.99] | 0.71 |
| Chaosuwannakit 2021 (20) | 0.47 [0.30; 0.63] | 0.60 | 0.98 [0.96; 0.99] | 0.70 |
| Phan 2019 (30) | 0.47 [0.30; 0.64] | 0.59 | 0.97 [0.95; 0.98] | 0.71 |
| Kim 2014 (31) | 0.48 [0.32; 0.65] | 0.60 | 0.97 [0.95; 0.98] | 0.65 |
| POOLED ESTIMATE | 0.47 [0.32; 0.62] | 0.56 | 0.98 [0.96; 0.99] | 0.67 |

Supplementary Table 4: Leave-one-out sensitivity analysis of non-contrast TTE studies

| Left-out Study | Sensitivity | | Specificity | |
| --- | --- | --- | --- | --- |
|  | Sensitivity [95% CI] | I^2^ | Specificity [95% CI] | I^2^ |
| Weinsaft 2016 (11) | 0.56 [0.43; 0.69] | 0.22 | 0.99 [0.96; 0.99] | 0.17 |
| Weinsaft 2009 (3) | 0.57 [0.41; 0.71] | 0.27 | 0.97 [0.95; 0.99] | 0.28 |
| Garg 2012 (19) | 0.64 [0.50; 0.76] | 0.00 | 0.98 [0.95; 0.99] | 0.35 |
| Kim 2014 (31) | 0.55 [0.42; 0.68] | 0.02 | 0.97 [0.94; 0.98] | 0.00 |
| POOLED ESTIMATE | 0.58 [0.46; 0.69] | 0.00 | 0.98 [0.96; 0.99] | 0.23 |

Supplementary Table 5: Leave-one-out sensitivity analysis of contrast TTE studies
