# Supplementary figures and images for: A systematic review and meta-analysis of transthoracic echocardiogram vs. cardiac magnetic resonance imaging for the detection of left ventricular thrombus
Source: Eur Heart J Imaging Methods Pract. 2023 Dec 7;1(2):qyad041. doi: 10.1093/ehjimp/qyad041 (PMC11240154; doi:10.1093/ehjimp/qyad041)

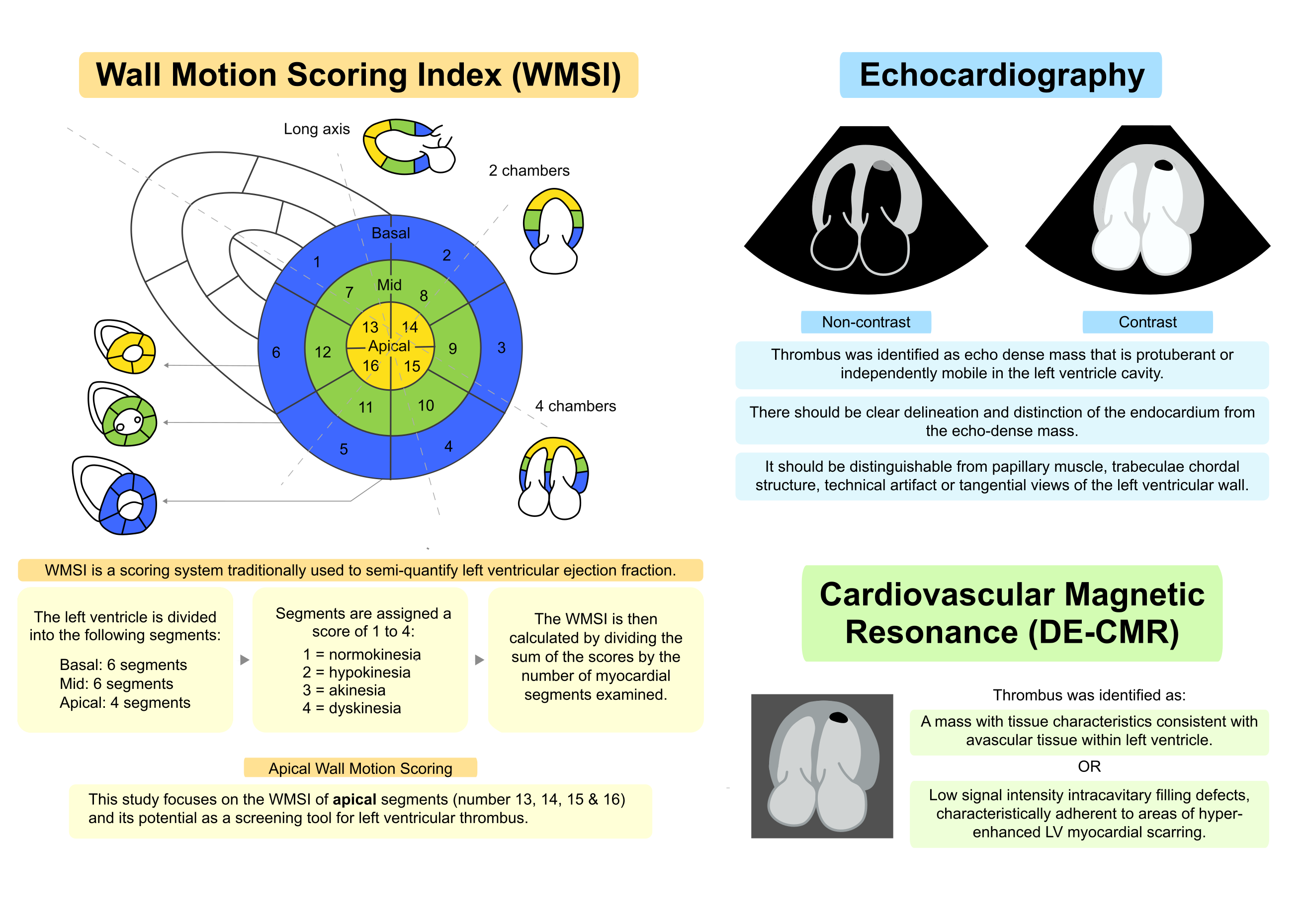

Supplement: qyad041_Supplementary_Data [file qyad041_Supplementary_Data.zip › Supplementary figure 1.tiff]

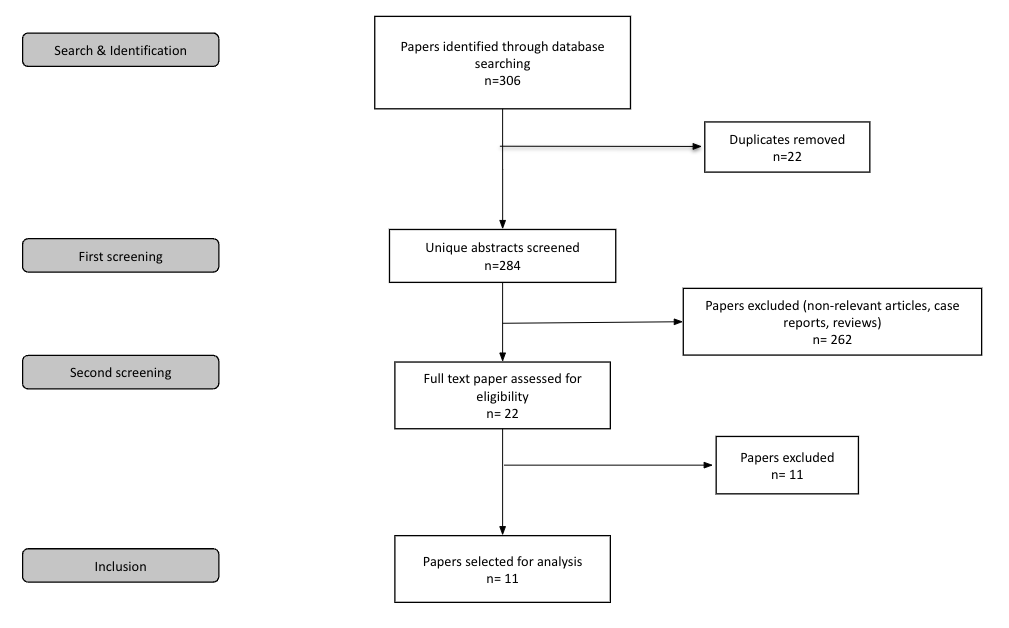

Supplement: qyad041_Supplementary_Data [file qyad041_Supplementary_Data.zip › Supplementary figure 2.tiff]

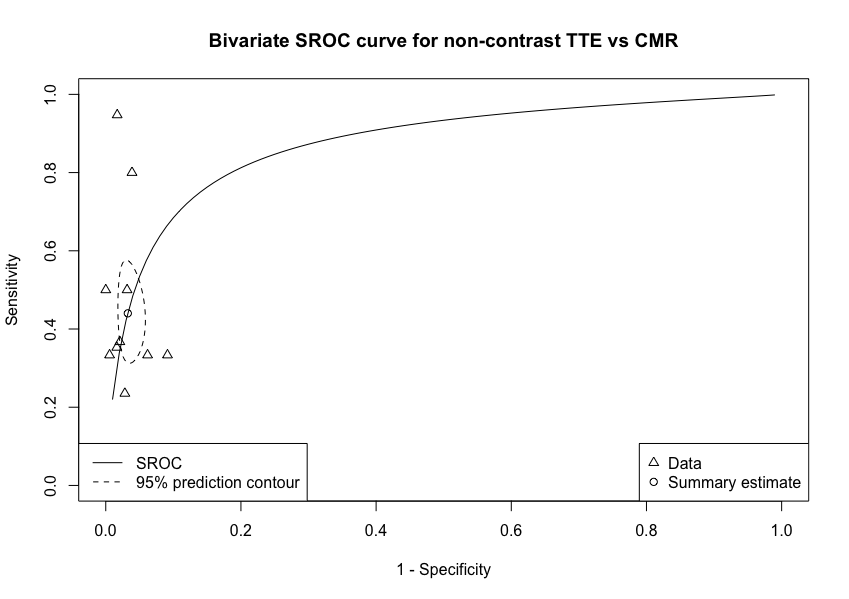

Supplement: qyad041_Supplementary_Data [file qyad041_Supplementary_Data.zip › Supplementary Figure 3a.tiff]

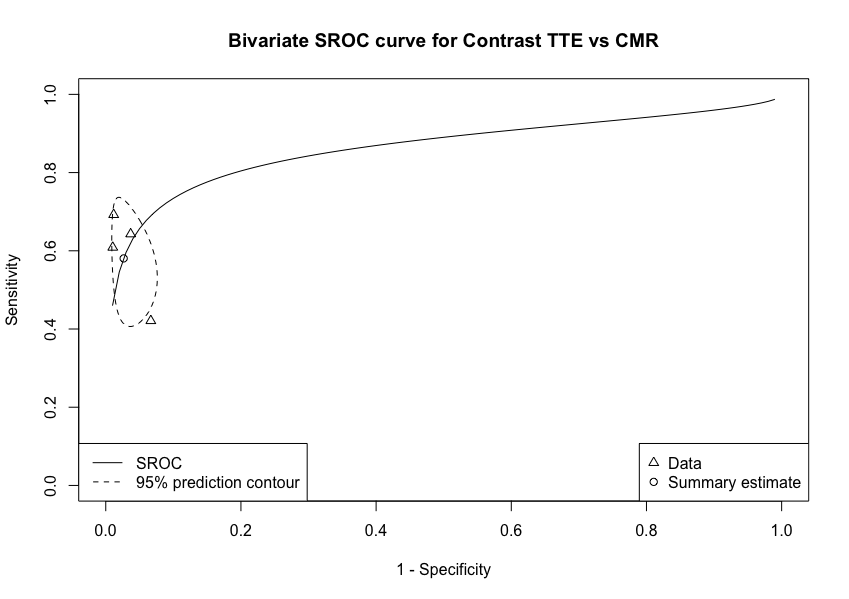

Supplement: qyad041_Supplementary_Data [file qyad041_Supplementary_Data.zip › Supplementary Figure 3b.tiff]

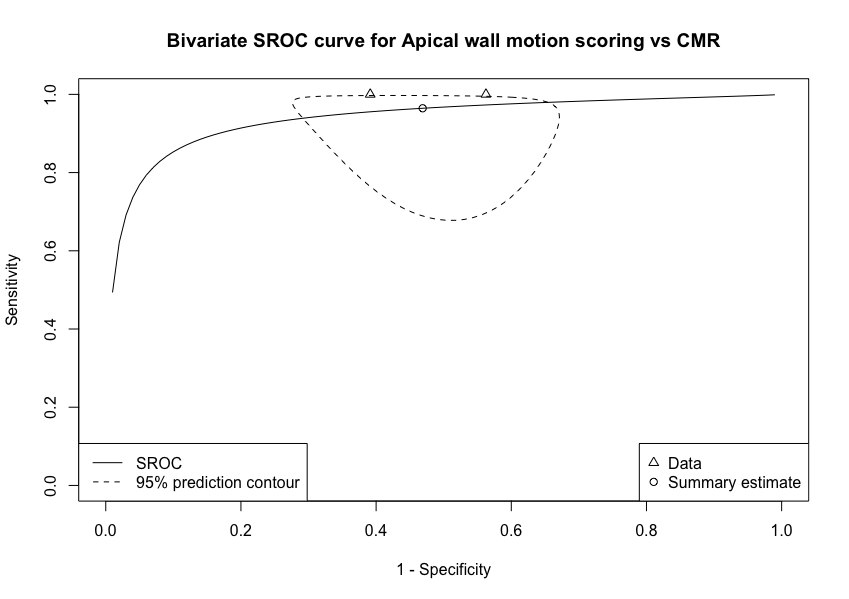

Supplement: qyad041_Supplementary_Data [file qyad041_Supplementary_Data.zip › Supplementary Figure 3c.tiff]
